# Supplementary material for: An efficient 3D column-only P300 speller paradigm utilizing few numbers of electrodes and flashings for practical BCI implementation
Source: PLoS One. 2022 Apr 12;17(4):e0265904. doi: 10.1371/journal.pone.0265904 (PMC9004785; doi:10.1371/journal.pone.0265904)
Supplement: S1 File — (DOCX) [file pone.0265904.s001.docx]

The dataset supporting the conclusions of this article is available in the Kaggle Database repository: <https://www.kaggle.com/onurerdemkorkmaz/3d-column-only-p300-speller-paradigm-dataset>
