# Supplementary material for: An efficient 3D column-only P300 speller paradigm utilizing few numbers of electrodes and flashings for practical BCI implementation
Source: PLoS One. 2022 Apr 12;17(4):e0265904. doi: 10.1371/journal.pone.0265904 (PMC9004785; doi:10.1371/journal.pone.0265904)
Supplement: Attachment — Submitted filename: Response to Reviewers.docx [file pone.0265904.s002.docx]

**AUTHOR RESPONSES TO THE REVIEWERS**

**Journal:** PONE-D-21-29800

**Title of the manuscript:** An efficient 3D column-only P300 speller paradigm utilizing few numbers of electrodes and flashings for practical BCI implementation

**Authors:** Onur Erdem Korkmaz, Onder Aydemir, Emin Argun Oral and Ibrahim Yucel Ozbek

We would like to start by thanking the editor(s) and the reviewers for their useful comments and suggestions on how to further improve the quality of my manuscript. I have been able to incorporate changes to reflect all the suggestions provided by the reviewer. Here is a point-by-point response to the reviewer’s comments and concerns.

**Reviewer 1:**

**1. Asked:**  Please amend the title either on the online submission form or in your manuscript so that they are identical.

**1. Answer:** We amended the titles both on the online submission form and in our manuscript.

**2. Asked:** Please explain the rationale for the development of your work, and clearly indicating which problem with existing approaches you are addressing. Also describe about the datasets used in this work briefly. Please clearly report at the beginning of your methods or results section which the key performance measures were to establish validity and utility of your new system.

**2. Answer:** We are very grateful to reviewer for his valuable comment, which helped us to improve the quality of our manuscript. Based on his/her suggestion we explain the important points, including the problem which we addressed, dataset and achieved performance, in the subsection of *The Proposed Method.* The added sentences are also given below.

In this study, we proposed a 3D column (3D-C) intensified based P300 speller system, which provided higher CA rate and lower user workload than the classical 2D-RC P300 speller paradigm. The EEG signals were recorded in both 3D and 2D paradigm procedures from ten healthy participants. In both procedures, 60 characters were presented to each participant during the data collection phase. The proposed 3D-C paradigm was successfully applied to the datasets and we achieved an average CA rate of 99.81% for binary classification (target-nontarget) and 99.2% character detection accuracy on the test data by the traditional two-layer artificial neural networks (ANN) model with a single output neuron.

Please see *The Proposed Method* section on page 4.

Moreover, we added a brief introduction at the beginning of The Results section as follows:

In this study, we proposed an efficient 3D column-only P300 speller paradigm utilizing few numbers of electrodes and flashings for practical BCI implementation. In this section, we provided the experimental results including P300 detection and the target character recognition performances achieved by the traditional two-layer ANN model with a single output neuron.

Please see the *Results* section on page 11.

**Reviewer 2:**

**1. Asked:** Figure 1 should be clearer.

**1. Answer:** Based on the reviewer’s suggestion, we improved the readability of Figure 1.

**2. Asked:** Data set references are needed.

**2. Answer:** We are very grateful to the reviewer pointing out this issue. The datasets presented in this study were used for the first time in the literature. Therefore, this paper will be cited by the researchers, who will use these datasets. On the other hand, in the section of Introduction, we cited other datasets which were presented by other researchers.

**Comment from the editor:**

**Asked:** The [PLOS Data policy](http://www.plosone.org/static/policies.action#sharing) requires authors to make all data underlying the findings described in their manuscript fully available without restriction, with rare exception (please refer to the Data Availability Statement in the manuscript PDF file). The data should be provided as part of the manuscript or its supporting information, or deposited to a public repository. For example, in addition to summary statistics, the data points behind means, medians and variance measures should be available. If there are restrictions on publicly sharing data—e.g. participant privacy or use of data from a third party—those must be specified.

**Answer:** We mentioned the Data Availability in the section of Acknowledgement as follows:

*On the other hand, the datasets, which were presented in this study, could be available for the researchers by contacting the corresponding author via e-mail.*

Please see the section of Acknowledgement on page 15.
