# Supplementary material for: An efficient 3D column-only P300 speller paradigm utilizing few numbers of electrodes and flashings for practical BCI implementation
Source: PLoS One. 2022 Apr 12;17(4):e0265904. doi: 10.1371/journal.pone.0265904 (PMC9004785; doi:10.1371/journal.pone.0265904)
Supplement: Attachment — Submitted filename: Response to Reviewers.docx [file pone.0265904.s003.docx]

**AUTHOR RESPONSES TO THE ACADEMIC EDITOR**

**Journal:** Plos One

**Title of the manuscript:** An Efficient 3D Column-Only P300 Speller Paradigm Utilizing Few Numbers of Electrodes and Flashings for Practical BCI Implementation

**Manuscript ID:** PONE-D-21-29800R1

**Authors:** Onur Erdem Korkmaz, Onder Aydemir, Emin Argun Oral and Ibrahim Yucel Ozbek

We would like to start by thanking the editor(s) and the reviewers for their useful comments and suggestions on how to further improve the quality of my manuscript. We have been able to incorporate changes to reflect all the suggestions provided by the academic editor. Here is a point-by-point response to the academic editor.

**Academic Editor:**

**1. Asked:**  Please check the title of the paper in the system.

### **1. Answer:** We checked the title of the paper in the system. We realized that while the first letters of the words were not written in capital letters in the manuscript, but they were given with capital letters in the system. Therefore, we edited the manuscript with capital letters.

**2. Asked:** There are typos in the article, please read the paper very carefully.

**2. Answer:** We read the paper very carefully and corrected the typos.

**3. Asked:** The font size in the figures must be same.

**3. Answer:** We set the font sizes same in the figures.

**4. Asked:** Also mention the impact and reproducibility of this work.

**4. Answer:** We mentioned the impact and reproducibility of this work at the end of the Conclusion section. The added statement is also given below:

We believe that the proposed method has a great potential to achieve higher performance for improving the BCI spelling systems. On the other hand, we make the entire dataset publicly available to the community to encourage the reproducibility of this work.
